# Self-Sensing of Damage Progression in Unidirectional Multiscale Hierarchical Composites Subjected to Cyclic Tensile Loading

**DOI:** 10.3390/s16030400

**Published:** 2016-03-18

**Authors:** J. J. Ku-Herrera, O. F. Pacheco-Salazar, C. R. Ríos-Soberanis, G. Domínguez-Rodríguez, F. Avilés

**Affiliations:** Centro de Investigación Científica de Yucatán A.C., Unidad de Materiales, Calle 43 No.130, Col. Chuburná de Hidalgo. C.P. 97200, Mérida, Yucatán, Mexico; jesuskuh@live.com.mx (J.J.K.-H.); pacheco.salazar140585@gmail.com (O.F.P.-S.); rolando@cicy.mx (C.R.R.-S.); gashdmi@gmail.com (G.D.-R.)

**Keywords:** hierarchical composites, carbon nanotubes, damage sensing, damage accumulation, cyclic loading

## Abstract

The electrical sensitivity of glass fiber/multiwall carbon nanotube/vinyl ester hierarchical composites containing a tailored electrically-percolated network to self-sense accumulation of structural damage when subjected to cyclic tensile loading-unloading is investigated. The hierarchical composites were designed to contain two architectures differentiated by the location of the multiwall carbon nanotubes (MWCNTs), *viz.* MWCNTs deposited on the fibers and MWCNTs dispersed within the matrix. The changes in electrical resistance of the hierarchical composites are associated to their structural damage and correlated to acoustic emissions. The results show that such tailored hierarchical composites are able to self-sense damage onset and accumulation upon tensile loading-unloading cycles by means of their electrical response, and that the electrical response depends on the MWCNT location.

## 1. Introduction

With the increased use of fiber-reinforced polymer composites (FRPCs) for structural applications, such as aerospace, marine, wind turbine, and automotive industries, the interest in developing structural health monitoring (SHM) techniques that ensure a safe structural performance of the composite has also increased [1]. Failure mechanisms of FRPCs are more complex than those of monolithic materials, such as metals or ceramics, given the contrasting mechanical properties between the matrix and fibers, the existence of an interface, the reinforcement orientation, and the manufacturing defects, to name a few [2]. Traditional SHM methods used for periodic inspections of composite structures, such as ultrasonics, X-ray radiography, infrared thermography, holographic interferometry, and eddy currents require extensive human involvement and expensive procedures, becoming more difficult to implement for *in situ* SHM [3]. A promising SHM approach that can overcome these issues consists in making the composite electroconductive in such a way that, for an applied stress/strain, the composite experiences a change in its electrical conductivity. An advantage of this SHM technique is that the composite itself is capable of tracking its own damage progression (*i.e.*, embedment of external sensors is not required) and hence, does not suffer from problems associated with stress concentrations (as for common embedded sensors). In addition, this method allows the entire structure to be monitored [4,5,6]. This technique also circumvents the issue of sensitivity to damage accumulation of acoustic emission (AE) by using the residual electrical resistance after unloading the structure. Electrical resistance measurements have been widely used to detect damage in carbon fiber-reinforced composites [5,7,8,9,10]. Due to their inherent electrical conductivity, breakage of the load-carrying carbon fibers result in changes in the composite electrical conductivity [5,7,8,9]. Many works have been conducted to improve the understanding and interpretation of this approach. For instance, Abry *et al.* [11] measured the longitudinal and transverse changes in electrical resistance of unidirectional carbon fiber/epoxy specimens loaded in tension; given material anisotropy, the electrical resistance response under tension depends also on the fiber orientation. The authors found that when the composite is loaded in tension along the fiber direction, the changes in electrical resistance are dominated by fiber breakage, while when it is loaded transversally, the electrical response is due to the loss of contacts among the adjacent fibers. To extend this concept to different loading scenarios, the group led by Deborah Chung monitored the electrical resistance changes for tensile monotonic and cyclic, flexural, and impact loadings in carbon fiber/epoxy composites [5,8,12]. Since then, significant research has been devoted to characterize and implement the electrical resistance approach using carbon fibers as reinforcements and self-sensing components [3,9]. Given the recent advances in nanotechnology and the commercial availability of nanomaterials with outstanding properties, they have been mixed with thermosetting polymers and infiltrated through fiber preforms to fabricate what has been named “multiscale hierarchical composites”. Carbon nanofibers [13], carbon black nanoparticles [14] and carbon nanotubes (CNTs) [4,6,15,16,17] are some examples of nanomaterials used to build such multiscale hierarchical composites. In the particular case of CNTs, they can form electrically-conductive networks within a vast number of thermosetting polymers at less than 0.5 wt. % [18]. Given the size difference between conventional micrometric fibers and carbon nanotubes (nanometric diameters), it is possible to place carbon nanotubes in matrix-rich areas among fibers as well as between adjacent plies [6,17,19]. The CNT network allocated within the matrix allows to track the evolution of damage of the composite by measuring the changes in its electrical resistance during mechanical deformation, showing remarkable sensitivity to matrix-dominated failure mechanisms [6]. Using this configuration (CNTs dispersed within the matrix) a number of works have been devoted to correlate the electromechanical response to the composite’s damage under different loading scenarios [4,15,16,20,21,22,23]. A different hierarchical configuration of CNT-based composites for self-sensing applications through electrical resistance consists in placing CNTs directly over the (non-conductive) fibers. Current research has reported the use of this technique and material architecture for single-fiber composites [24,25,26], but the technique needs to be extended to more realistic configurations as composite laminates. Given this motivation, this work investigates the capability of multiwall carbon nanotube (MWCNT)/glass fiber/vinyl ester composites with a tailored electrical MWCNT network to self-sense composite damage initiation and accumulation when they are subjected to tensile loading-unloading cycles; acoustic emission is used as a benchmark technique to validate the correlation between the electrical resistance variations and composite’s damage. In order to tailor the composite’s electrical sensitivity, the multiscale hierarchical composites are manufactured into two architectures differentiated by location of the MWCNTs: (i) with MWCNTs randomly dispersed within the matrix, and (ii) with MWCNTs deposited onto the glass fibers.

## 2. Materials and Methods

### 2.1. Materials

Commercial E-glass fibers (Poliformas Plásticas S.A de C.V. Mérida, Mexico) with an average diameter of 15 μm, density of 2.54 g/cm^3^ in the form of fiber tows containing ~4000 filaments per tow were used as unidirectional fibers. Commercial MWCNTs (Cheap Tubes Inc., Cambridgeport, VT, USA) with purity >95%, 30–50 nm outer diameter, 5–10 nm inner diameter, and 1–6 μm length range were used. All MWCNTs used were chemically oxidized using a solution of H_2_SO_4_/HNO_3_ at 3.0 mol/L for 2 h, following the procedure reported in [27]. An epoxy vinyl ester Hetron 992 FR resin from Ashland composites (Dublin, OH, USA) was used as the thermosetting matrix for composite manufacturing. Cobalt naphthenate (CoNap) in a proportion of 0.2 wt. % and 0.6 wt. % of methyl ethyl ketone peroxide (MEKP) were employed to manufacture the composites.

### 2.2. Composite Manufacturing

**Figure 1:**
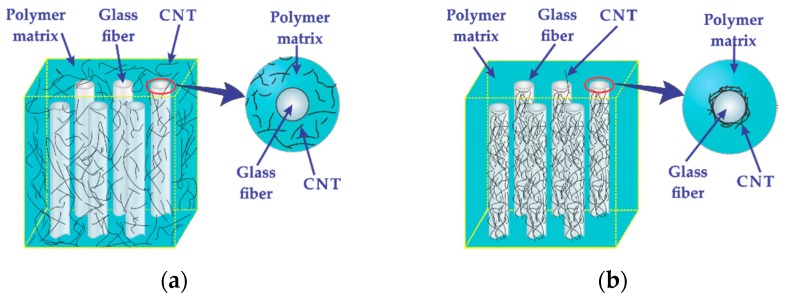
Hierarchical composite architectures. (**a**) Composite architecture *m*, with MWCNTs dispersed within the matrix, and (**b**) composite architecture *f*, with MWCNTs deposited onto the fibers.

To tailor the composite’s electrical sensitivity, the multiscale hierarchical composites were manufactured into two architectures depending on the location of the MWCNTs: (i) with MWCNTs randomly dispersed within the matrix, and (ii) with MWCNTs deposited onto the glass fibers. These multiscale composites will be named hereafter as architecture “*m*” for composites containing MWCNTs randomly dispersed within the matrix (Figure 1a), and architecture “*f*” for those where the MWCNTs are deposited onto the glass fibers (Figure 1b). The deposition of oxidized MWCNTs onto the glass fibers was conducted following the procedure reported in our previous works [28,29,30]. Briefly, oxidized MWCNTs are first ultrasonically dispersed in water, and then glass fiber tows are immersed into the MWCNT/water mixture, assisted by ultrasonic agitation. The glass fiber tows containing MWCNTs are then extracted from the container and dried in an oven.

**Figure 2:**
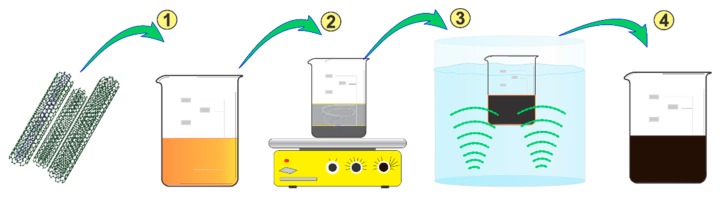
Procedure used to disperse MWCNTs within the vinyl ester resin prior to resin infusion.

To manufacture the hierarchical composites, a layup consisting of three layers of 14 cm long glass fibers was used. For the composite with architecture *m*, the preform was made of as-received glass fibers while, for architecture *f*, the preform was made using glass fibers with deposited MWCNTs. Both composite architectures depicted in Figure 1 were manufactured by vacuum-assisted resin transfer molding. For composites with architecture *m*, a modified matrix with dispersed MWCNTs was used to impregnate the glass fiber preform. Such a MWCNT-modified matrix was achieved by mixing 0.5 wt. % of MWCNTs with the vinyl ester resin prior to infusion. The dispersion of MWCNTs within the resin was conducted as indicated in Figure 2. This procedure consisted in adding 0.5 g of oxidized MWCNTs into 100 g of vinyl ester (1) and mixing them by mechanical stirring for 1 h (2) followed by dispersion aided by an ultrasonic bath operated at 42 kHz and 70 W for 3 h (3). The MWCNT-modified vinyl ester (4) was used to impregnate the preform made of as-received glass fibers.

For the composite architecture *f*, a conductive MWCNT-modified vinyl ester with a concentration of 0.5 wt. % MWCNTs was applied only at the ends of the fiber preform, to promote electrical contact among fibers and to consolidate the electrodes. Then the glass fiber preform with the defined electrodes was impregnated with neat vinyl ester resin (without MWCNTs) by resin infusion. For both composite architectures, *f* and *m*, the vinyl ester resin (neat or MWCNT-modified) was infused into the fiber preform assisted by vacuum at a rate of ~10 mL/min. The laminate was left for curing at room temperature for 2 h and then taken out of the mold for post-curing at 82 °C for 4 h in a convection oven.

### 2.3. Specimens Preparation and Test Setup

**Figure 3:**
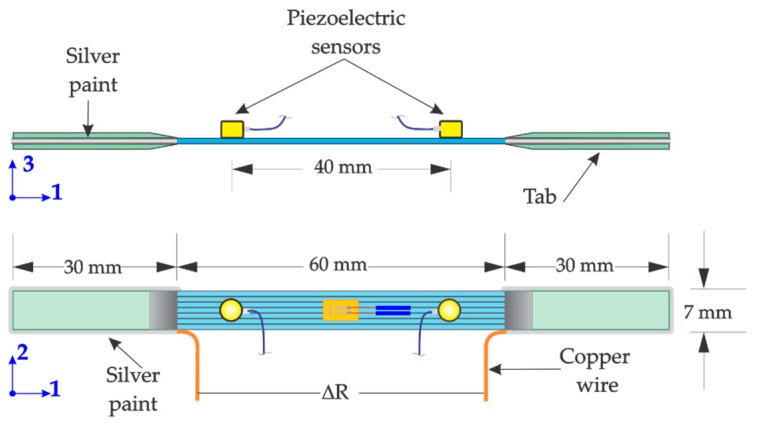
Tensile test specimen dimensions and instrumentation for *in situ* electrical monitoring coupled with acoustic emission.

The changes in electrical resistance of the hierarchical composites were measured using unidirectional (0°) laminates with the fibers aligned with the loading direction. The specimens’ instrumentation for the electromechanical characterization involved tabbing the laminates, bonding strain gages, and electrode instrumentation, as schematized in Figure 3. In this figure the conventional (1,2,3) material coordinate system is used to indicate the fiber (1), in-plane transverse (2), and through-thickness (3) directions. Tensile specimens were obtained from the unidirectional laminates with dimensions scaled down (ratio of 1:2) from the dimensions recommended by the ASTM standard D3039 for 0° specimens [31]. The specimens were 120 mm long and 7 mm wide, with a thickness of ~1.0 mm defined by the three plies employed. The 25 mm long tabs were made of plain wave glass fiber/vinyl ester laminates, and were adhesively bonded to the specimen ends. The electrical resistance of the specimens was measured from a pair of copper wires running around the periphery of the tabs bonded to the specimen with silver paint. In order to calculate the volume electrical conductivity (*σ_e_*) of the hierarchical composites, their electrical resistances without loading (*R*_0_) was measured before tensile testing.

A Shimadzu AG-I (Shimadzu, Kyoto, Japan) universal testing machine equipped with a 20 kN load cell was employed for all tests. The crosshead displacement rate of the universal testing machine was 1 mm/min. The specimens’ strain was recorded by means of unidirectional strain gages (350 Ω, gage factor of 2.125) using a Vishay P3 strain indicator. The electrical resistance (*R*) of the specimen was measured in real-time during the tests using an Agilent DMM 3441A digital multimeter, synchronizing all instruments via an in-house data acquisition software. For the AE analysis, two PICO-type piezoelectric transducers were attached onto the specimen´s surface leaving a measurement span of 40 mm, as depicted in Figure 3. The two PICO-type sensors were connected to a PCI-2-based AE system (Physical acoustic, Princeton Junction, NJ, USA) to acquire the acoustic events during the tensile test. A threshold of 40 dB was used to filter-out the noise not related to the acoustic events within the specimen. Additionally, all acoustic events not coming from the gap between sensors were discarded by data post-processing. Five replicates for each composite architecture were tested. The axial stress (*σ*), strain (*ε*), the electrical resistance, and the acoustic events occurring within the specimens were acquired simultaneously. Cyclic loading-unloading tests were conducted in order to investigate damage accumulation. A total of six incremental loading-unloading tension cycles were applied to the specimens controlled by the maximum applied load. These load levels sequentially reached maximum forces of *F_max_* = 0.5, 1.0, 1.5, 2.0, 2.5, and 3.0 kN, and were carefully chosen to cover the mechanical response of the composite from the elastic region until evident damage is detected by AE. The change in electrical resistance (∆*R* = *R* − *R*_0_) normalized by the electrical resistance of the load-free state (*R*_0_), the stress (*σ*), and the acoustic events were plotted as function of the strain (*ε*) for each loading-unloading cycle. Additionally, for two selected specimens, 15 additional loading cycles up to a maximum force of 3.0 kN were applied to the same specimen that was previously loaded under the six incremental cycles discussed earlier. These experiments allowed to investigate damage accumulation due to cyclic loading-unloading conditions.

## 3. Results and Discussions

### 3.1. Electrical Conductivity of the Hierarchical Composites

The composites’ electrical conductivity was characterized along the fiber direction. The electrical conductivity of the composites was obtained by measuring the electrical resistance of the specimens used for the tensile test free of any mechanical load (*R*_0_) and using the specimen’s gage length (~60 mm) and cross-sectional area (~7 mm^2^). The composites with architecture *m* exhibited a mean volumetric electrical conductivity of 3.43 × 10^−3^ S/m (with a standard deviation of ±1.03 × 10^−3^ S/m), while for the composites with architecture *f* the electrical conductivity was 1.70 × 10^−1^ ± 4.00 × 10^−3^ S/m. The higher electrical conductivity of the composite with architecture *f* results from the contribution of electrically-conductive pathways created along the fibers upon MWCNT deposition and due to additional lateral contacts among adjacent fibers of the laminate.

### 3.2. Damage Progression under Monotonic Tensile Loading

**Figure 4:**
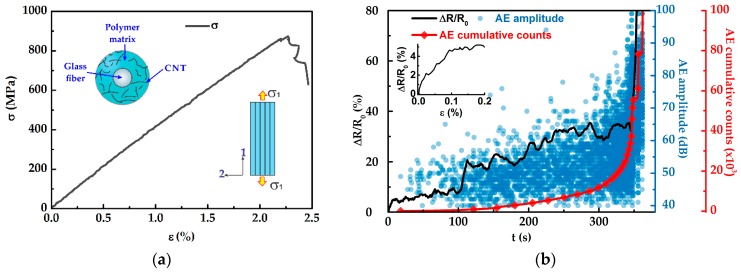
Mechanical, electromechanical, and acoustic emission characterization of the composite with architecture *m*. (**a**) Stress-strain curve, (**b**) evolution of ∆*R*/*R*_0_ (continuous line), AE amplitude (circles), and AE cumulative counts (diamonds) during the tensile test.

Figure 4 plots the results of the mechanical, electromechanical and AE characterization of a representative composite with architecture m subjected to monotonic tensile loading up to failure. A representative *σ*-*ε* curve is shown in Figure 4a while the electrical (Δ*R*/*R*_0_) and AE signals (amplitude and cumulative counts, circles, and diamonds, respectively) as a function of elapsed time (*t*) are shown in Figure 4b. As seen from Figure 4a, the composites with architecture *m* exhibit a linear trend for almost the whole stress-strain curve, and a drop of stress is observed close to failure, portraying the loss of load-bearing capacity. As seen in Figure 4b, during the firsts 20 s of the test (corresponding to *ε* < 0.2% in Figure 4a) no acoustic events are detected, suggesting that the Δ*R*/*R*_0_ response measured is due to elastic deformation of the composite, *i.e.*, piezoresistivity (see inset in Figure 4b). In this region the tensile strain applied to the composite equally stretches the matrix and fibers, modifying the separation among MWCNTs and resulting in an increase in the electrical resistance of the composite. At *t* ~20 s the first acoustic events are detected, indicating the onset of composite damage. Thereafter, the number of acoustic events progressively increase as the specimen is loaded up to failure. Analysis of the amplitude of the acoustic events [32,33] suggests that the composite´s damage is generated by a combination of three sequential damage mechanisms, *viz.* matrix microcracking, fiber/matrix debonding, and fiber breakage. For low levels of strain (*ε* < 1.0%) the damage is mainly attributed to matrix microcraking as indicated by the amplitudes of the AE events lower than 60 dB (*t* < 170 s in Figure 4b) [32,33]. In addition to the acoustic events associated to matrix cracking (60–40 dB) for 20 < *t* < 170 s some acoustic events with amplitudes of 60–70 dB are detected, which are associated to fiber/matrix debonding [32,33]. In this region, the initiation and propagation of matrix cracks destroy the conductive pathways of the MWCNT network, which is reflected in the Δ*R*/*R*_0_ response exhibiting some oscillations with an overall increase in resistance. The oscillations observed in ∆*R*/*R*_0_ may arise from occurrence of unstable matrix cracks. The acoustic events associated to fiber/matrix debonding increase for *t* > 170 s (*ε* > 1.0%) until the specimen’s collapse. The onset of fiber breakage is expected around *t* ~ 170 s (*ε* ~ 1.0%), as indicated by a few acoustic events with amplitudes >70 dB [33,34,35]. For larger strains the occurrence of fiber breakage increase until specimen’s collapse. However, for the composite with architecture m, Δ*R*/*R*_0_ does not clearly reflect fiber breakage until imminent collapse. Since for this configuration, the MWCNTs are dispersed within the matrix, the sensitivity of the composite with architecture m to fiber breakage is relatively poor. For this composite architecture, such a signal may stem from cracking of matrix layers surrounding the fibers, which is triggered by fiber breakage.

**Figure 5:**
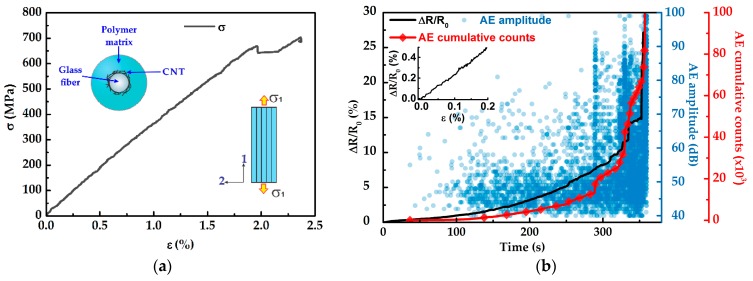
Mechanical, electromechanical, and acoustic emission characterization of the composite with architecture *f*. (**a**) Stress-strain curve, (**b**) evolution of ∆*R*/*R*_0_ (continuous line), AE amplitude (circles), and AE cumulative counts (diamonds) during the tensile test.

Results of the mechanical and electromechanical behaviors of the composite with architecture *f* are shown in Figure 5. Similar to the composite with architecture *m* under monotonic tensile loading, the composite with architecture *f* shows a linear stress-strain response for almost the whole curve, and a drop in stress is observed close to failure. For low levels of strain (*ε* < 0.2%, *t* < 25 s), no acoustic events are detected, suggesting that the structural integrity of the composite is still intact and hence, the ∆*R*/*R*_0_ signal arises purely from piezoresistivity (see inset in Figure 4b). The piezoresistive response of the composite with architecture *f* is originated from the deformation of the MWCNT network located at the fiber/matrix interface as a consequence of the load transfer from the matrix to the fibers. At *ε* ~0.2% the onset of composite damage occurs as suggested by the few acoustic emissions (40–60 dB) detected at *t* ~25 s. The main damage mechanism for low strain levels (*ε* < 1.0%) is attributed to matrix microcracking, as indicated by the acoustic events with amplitudes lower than 60 dB (*t* < 170 s in Figure 5b) [32]. Different to composites with architecture *m*, composites with architecture *f* show a smoother trend in ∆*R*/*R*_0_ with minimal oscillations. This is associated to a lower damage sensitivity of such composites to matrix cracking, given that MWCNTs are located onto the fibers in such a particular material architecture. Furthermore, the overall change in electrical resistance of composites with architecture *f* (Figure 5b) is smaller than that of composites with architecture *m* (Figure 4b); this is also likely because of increased damage sensitivity to matrix cracking for architecture *m*. Damage associated to fiber/matrix debonding is also detected for *ε* < 1.0% (*t* < 170 s) as indicated by acoustic emissions with amplitudes of 60–70 dB [32,33]. In Figure 5b the first acoustic events with amplitudes >70 dB (related to fiber breakage [33,34,35]) are detected at *t* ~ 160 s (*ε* ~ 1%). Thereafter, the ∆*R*/*R*_0_ response exhibits few sudden peaks, which coincides with important changes in the cumulative counts of the AE signals. In fact, the shape of the ∆*R*/*R*_0_ curve outstandingly follows that of the AE cumulative counts, pinpointing the high sensitivity of the electrical signal to detect composite damage. It is assumed that when the fibers break at those strain levels, fiber breakage is accompanied by more damage within the composite in the form of matrix cracking and fiber/matrix debonding (see amplitudes of the AE in Figure 5b). Above *t* > 160 s, ∆*R*/*R*_0_ increases sharply indicating continued fiber breakage. At *t* ~330 s (*ε* ~ 2.0%) an abrupt change in ∆*R*/*R*_0_ is observed, suggesting that the composite experiences severe damage associated to fiber breakage. However, at such a strain level the composite still maintains limited load bearing capacity since the load is redistributed as the fibers continue breaking; final collapse of the specimen occurs at *t* ~360 s.

### 3.3. Damage Accumulation

**Figure 6:**
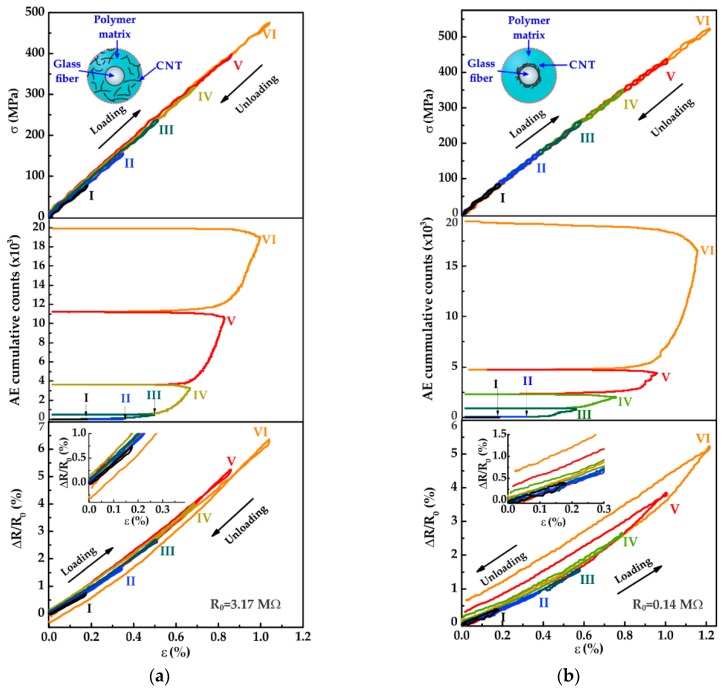
Coupled mechanical (**Top**), AE (**Middle**), and electromechanical (**Bottom**) responses of hierarchical composites under incrementally-increasing cyclic tensile loading. (**a**) Composite architecture *m*, (**b**) composite architecture *f*.

The unidirectional hierarchical composites were subjected to incrementally increasing cyclic tensile loading in order to investigate the electrical sensitivity of such composites to damage accumulation. The applied load was gradually increased in each cycle reaching a maximum strain of *ε* ≈ 1.2%, which is about half the failure strain seen in Figure 4a and Figure 5a. Figure 6a shows the mechanical (top), AE cumulative counts (middle), and the electromechanical (bottom) responses of a representative composite with architecture *m* subjected to incrementally increasing cyclic tensile loading. In Figure 6a the load level in cycle I (*F_max_* = 0.5 kN) was chosen to ensure that the composite’s integrity is intact. As seen from this figure the mechanical behavior is linearly elastic during the first loading-unloading cycle and no evidence of damage is detected by AE during this first cycle (Figure 6a, middle). The electromechanical response (Figure 6a, bottom) for this first cycle is also linear with the applied strain during the whole cycle and does not show residual (permanent) changes of electrical resistance after unloading, *i.e.*, after the first cycle ∆*R* = *R* − *R*_0_ = 0 as seen in the inset of Figure 6a (bottom). A very similar scenario regarding *σ* − *ε*, AE, and ∆*R*/*R*_0_ occurred for cycle II (*F_max_* = 1.0 kN), indicating that the composite was yet in the linear elastic regime. For cycle III (*F*_max_ = 1.5 kN), the level of applied load induced limited damage to the composite, which is evidenced by few acoustic events. At this cycle, although the permanent change in electrical resistance is yet small (∆*R* ≈ 0), the ∆*R*/*R*_0_ vs *ε* curve loses linearity, which is associated to damage initiation, likely by matrix cracking and/or matrix viscoelastic phenomena. By applying a higher load level in cycle IV (*F_max_* = 2.0 kN), the cumulative AE counts evidently increase up to ~4000 acoustic events. In this cycle, composite damage increased given the higher load level, and the ∆*R*/*R*_0_ response followed a nonlinear trend; after unloading ∆*R*/*R*_0_ shows only a modest permanent change (∆*R*/*R*_0_ ≈ 0.2%). For cycle V (*F*_max_ = 2.5 kN), the cumulative acoustic events markedly increase since at this load level matrix cracks are expected to propagate, inducing fiber/matrix debonding. For this higher load level the permanent ∆*R*/*R*_0_ after unloading is still small, but the unloading path of ∆*R*/*R*_0_ is slightly different to that of the loading one, indicating certain hysteresis. This hysteresis is attributed to the presence of irreversible phenomena (damage) in the matrix and probably at the fiber/matrix interface. Such a hysteresis may be driven by the release of residual stresses from the curing process [36] and/or matrix viscoelastic phenomena [37]. For the last cycle (cycle VI, *F_max_* = 3.0 kN), the AE cumulative counts indicate significant matrix and fiber/matrix damage which is also reflected in the ∆*R*/*R*_0_ response by not returning to zero upon unloading. It is important to notice that the permanent value attained by ∆*R*/*R*_0_ after the VI^th^ cycle is negative. This irreversible (negative) value of ∆*R*/*R*_0_ upon unloading was observed for the three tested specimens and is associated to matrix dominated processes, such as matrix yielding and viscoelasticity [37]. Releasing of curing stresses at the fiber/matrix interface could also be a contributing factor for this irreversible ∆*R*/*R*_0_ [36]. Since, in this composite the MWCNTs are dispersed within the matrix, the ∆*R*/*R*_0_ response is very sensitive to events occurring in such a polymer matrix. Limited fiber breakage is also expected during cycle VI and is correlated to a few acoustic events with amplitudes >70 dB; however, for this composite architecture (*m*) ∆*R*/*R*_0_ does not conspicuously reflect the onset of fiber breakage, since the MWCNTs in this composite are randomly located within the matrix, rather than onto the fibers.

The corresponding results of the composites with architecture *f* are shown by a representative plot in Figure 6b. As for the mechanical response of the composites with architecture *m*, the composites with architecture *f* behaves linearly elastic during cycles I and II (*F_max_* = 0.5 and 1.0 kN). The lack of detected acoustic events during cycles I and II indicates that the structural integrity of the composite remains intact during those cycles. The electromechanical responses of the two first cycles (Figure 6b, bottom) is also linear with the applied strain during the loading-unloading cycles, without any permanent change in electrical resistance upon unloading (see inset in Figure 6b, bottom). For cycle III (*F*_max_ = 1.5 kN), the applied load induces marginal matrix cracking to the composite, as confirmed by a few acoustic events with amplitudes of 40–60 dB (Figure 6b, middle). Despite that according to the acoustic events matrix cracking occurs during cycle III, ∆*R*/*R*_0_ returns to zero upon unloading. This is because at this stress level, damage occurs at the matrix level and the MWCNTs in this configuration are located onto the fibers. Notice that for cycle III ∆*R*/*R*_0_ loses its linear trend for the last part of the curve, which can be associated to the onset of matrix damage. By applying a higher load level (*F_max_* = 2.0 kN) in cycle IV, the accumulated acoustic events increase up to ~2500 events. In this cycle, composite damage increases with increased load and ∆*R*/*R*_0_ follows a nonlinear trend. After unloading, ∆*R*/*R*_0_ shows only a small permanent change (~0.2%) and an evident hysteresis in the curve, suggesting damage accumulation. At this loading level, it is expected that matrix cracking reaches the fiber/matrix interface (AE events of 60–70 dB [33]) which modifies the tailored MWCNT network in such a region. In cycle V, the cumulative AE signal doubles reaching ~5000 events at the maximum load. At this load level matrix cracks are expected to propagate inducing significant damage associated to fiber/matrix debonding, as previously discussed. In this cycle the electromechanical response shows an evident permanent ∆*R*/*R*_0_ of ~0.35%, in addition to the more pronounced hysteresis seen from the unloading curve of ∆*R*/*R*_0_ (Figure 6b, bottom). The destruction of the MWCNT conductive pathways at the fiber/matrix interface region is probably induced by the propagation of fiber/matrix debonding. In cycle VI, a pronounced increase of the accumulated acoustic events evidence the onset of fiber breakage (70–100 dB, amplitudes not shown in the plot). For this composite architecture (*f*) a positive permanent ∆*R*/*R*_0_ of ~0.65% is attained upon unloading cycle VI as showed in Figure 6b (bottom). This behavior contrasts that of the composite with architecture *m*, where the permanent change in electrical resistance upon unloading is negative. These contrasting electromechanical behaviors between the composites with architectures *f* and *m* are due to the deliberately different location of the MWCNT network in such composites, which is differently affected depending on the composite’s damage mechanism.

**Figure 7:**
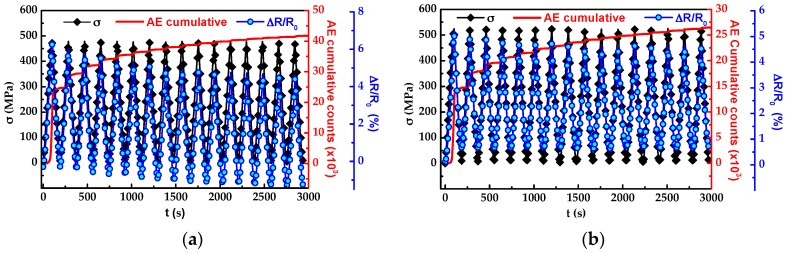
Evolution of stress (diamonds), ∆*R*/*R*_0_ (circles) and cumulative AE (red line) signals for a selected specimen subjected to cyclic loading. (**a**) Composite with architecture *m*, and (**b**) composite with architecture *f*.

After the sixth cycle shown in Figure 6, the same specimen was additionally subjected to 15 identical loading-unloading cycles. Figure 7 shows the evolution of stress (*σ*, diamonds), ∆*R*/*R*_0_ (circles) and the cumulative acoustic events (continuous red line) for both composite architectures, *m* (Figure 7a) and *f* (Figure 7b). To better interpret this analysis, the cumulative acoustic events and ∆*R*/*R*_0_ were reset to zero for the first cycle presented in Figure 7 (which correspond to the last cycle (VI) in Figure 6), and the new cumulative acoustic events were associated to accumulation of damage in the composite due to these new repetitive loading-unloading cycles at a constant stress level. For all cycles, the specimens reached a maximum stress of 500 MPa and returns to *σ* = 0 upon unloading.

During the loading segment of the first cycle, no acoustic events are detected until *σ* ≈ 400 MPa, which corresponds to the maximum stress in cycle V, see Figure 7a. Then the acoustic events accumulate ~25 × 10^3^ counts at the maximum stress level (500 MPa). For the loading segment of the first cycle, ∆*R*/*R*_0_ increases up to 6.2% at the maximum stress level and decreases to ~−0.3% upon unloading. For the second cycle in Figure 7a, a lower number of acoustic events are detected compared to the first one, given that a higher load level is necessary to generate more damage in the composite. For this second cycle, ∆*R*/*R*_0_ increases during loading and its maximum value was ~5.9%, a bit lower than that of the first cycle. For the unloading segment, again ∆*R*/*R*_0_ decreases as the load was released but in this case it reaches ~−0.43% upon full unloading. After each loading-unloading cycle, the amplitude of the ∆*R*/*R*_0_ response reduces progressively, exhibiting negative permanent values upon unloading. This behavior is associated to matrix yielding and viscoelastic matrix-dominated phenomena [37] and is consistent to that observed in Figure 4 for the composite architecture *m*.

The corresponding loading excursions for architecture *f* are shown in Figure 7b, where the additional 15 identical loading-unloading cycles of constant applied stress of 500 MPa are indicated by the curve representing *σ*. The evolution of *σ* (diamonds), ∆*R*/*R*_0_ (circles), and the cumulative AE (continuous red line) over time are shown in Figure 7b. During the loading segment of the first cycle, no acoustic events are detected until *σ* ≈ 400 MPa; then the acoustic events accumulate ~15 × 10^3^ counts for *σ* = 500 MPa. After the loading segment of the first cycle, ∆*R*/*R*_0_ increases up to ~5.1% at the maximum stress level, and decreases to ~0.6% when the specimen is completely unloaded. For the second cycle, fewer acoustic events are detected compared to the first cycle, since a higher stress level is required to generate more damage in the specimen. For the second cycle, again ∆*R*/*R*_0_ increases during loading, but in this case ∆*R*/*R*_0_ only reached ~4.8% at the maximum stress level, and ~0.6% upon unloading. In contrast with the composite with architecture *m*, the ∆*R*/*R*_0_ response of the composite with architecture *f* does not show negative values upon unloading and there are no increments in the permanent change in electrical resistance upon unloading. The reduction in amplitude of the ∆*R*/*R*_0_ response in this composite is associated to fiber/matrix debonding, since during the loading-unloading cycles the applied load propagates the existing cracks through the fiber/matrix interface region, decreasing the matrix-to-fiber load transfer efficiency. It is also likely that after each cycle, a few fibers break which can also contribute to the decrease in amplitude of the ∆*R*/*R*_0_ signal.

**Figure 8:**
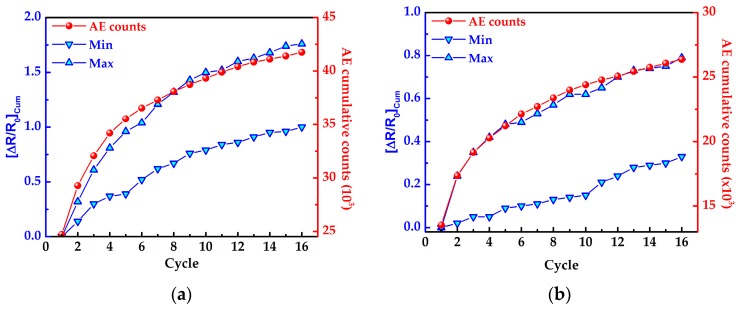
Cumulative ∆*R*/*R*_0_ response (Equation (1)) for assessment of damage accumulation under identical loading cycles. (**a**) Composite with architecture *m*, and (**b**) composite with architecture *f*.

The fact that the maximum (peak at *σ_max_* = 500 MPa) and minimum (valley at *σ* = 0) values of ∆*R*/*R*_0_ may be used as a metric of damage accumulation can be better assessed with the aid of Figure 8. Such a figure plots the accumulated difference (absolute value) between subsequent peak (labeled “Max”) values of ∆*R*/*R*_0_ as a function of the cycle number (*k*) which is quantified by:

(1) $${\left[\Delta R/R_{0}\right]}_{Cum}=\sum_{i=1}^{k-1}\left|{\left[\Delta R/R_{0}\right]}_{i}^{Max}-{\left[\Delta R/R_{0}\right]}_{i+1}^{Max}\right|$$

Equation (1) is stated in terms of the maximum values of ∆*R*/*R*_0_ but is also valid for the minimum ones (valleys, labeled “Min”). Both values, “Max” and “Min” are plotted in Figure 8. As seen from this figure, for composite architecture *m* (Figure 8a) both accumulated electrical curves (“Max” and “Min”) keep a close correlation with the cumulative AE, being the accumulated values of ∆*R*/*R*_0_ corresponding to the peak values more sensitive. The “Max” curves of both composite architectures correlates better with the damage accumulation detected by AE probably because at the peak stress the microcracks open, while they tend to close when the stress returns to zero. For composite architecture *f*, a close correlation between the electrical and AE cumulative counts is also observed but only for the maximum values of ∆*R*/*R*_0_. As inferred from the numerical values of the vertical axis in Figure 8a,b the composites with architecture *m* are more sensitive to this kind of damage accumulation than those with architecture *f*. This again, reveals that the tailored architecture of the MWCNT network within the composite has a paramount effect on its sensitivity to damage accumulation. At the repeated load levels experienced in Figure 8 (500 MPa, see Figure 7) matrix damage (rather than fiber damage) is expected, which is consistent with the location of the MWCNTs in architecture *m*.

## 4. Conclusions

The electrical capability of glass fiber/carbon nanotube/vinyl ester unidirectional hierarchical composites containing a tailored electrical network of multiwall carbon nanotubes (MWCNTs) to self-sense their damage progression under cyclic loading was investigated. Tailored MWCNT networks were achieved by deliberately placing the MWCNTs either randomly dispersed within the polymer matrix (architecture *m*) or deposited onto the glass fibers (architecture *f*). By using incrementally-increasing cyclic tensile loading tests, damage initiation and progression were identified by acoustic emission (AE) and correlated to *in situ* measurements of the composite´s electrical resistance. Cyclic loading-unloading tests where the maximum applied stress was gradually increased and cyclic tests where the maximum stress was kept constant for 15 more cycles were conducted. For both composite architectures, the onset of matrix cracking was identified by a deviation of linearity in the Δ*R*/*R*_0_ vs *ε* curve. For the composites with MWCNTs dispersed within the matrix, ∆*R*/*R*_0_ returns to zero upon unloading unless a critical stress is reached where composite damage initiates, which correlates well with the acoustic events. A negative permanent change of electrical resistance upon unloading characterized damage accumulation of the composite with MWCNTs dispersed within the matrix; this was associated to matrix-dominated irreversible phenomena such as yielding and viscoelasticity. On the other hand, the composites with MWCNTs deposited onto the fibers showed a positive permanent change in electrical resistance upon unloading, which was associated to fiber/matrix debonding and fiber breakage. The multiscale composites with MWCNTs on the fibers were able to detect the onset of composite damage by a deviation from linearity and hysteresis in the electromechanical curve, as well as to detect damage progression, being very sensitive to fiber-dominated damage mechanisms. The accumulation and progression of matrix damage due to repetitive loading-unloading cycles at a peak stresses of 500 MPa were tracked by both composite architectures, being the composite with MWCNTs dispersed in the matrix more sensitive to this kind of damage accumulation. The different electrical responses of the composites with architectures *m* and *f* when they are subjected to cyclic tensile loading highlights the tailored sensitivity to damage in such multiscale hierarchical composites. Therefore, the multiscale hierarchical composites developed in this study are excellent candidates for health monitoring of structures subjected to cyclic loading, and their sensitivity can be tailored for specificity from their hierarchical structure regarding the MWCNT location within the composite.
